# Supplementary material for: Genetic architecture of telomere length in 462,666 UK Biobank whole-genome sequences
Source: Nat Genet. 2024 Aug 27;56(9):1832–40. doi: 10.1038/s41588-024-01884-7 (PMC11387196; doi:10.1038/s41588-024-01884-7)
Supplement: Supplementary file 1 — Supplementary Figs. 1–15, Tables 1–19, Methods and Notes 1–4. [file 41588_2024_1884_MOESM1_ESM.pdf]

nature genetics

Supplementary information https://doi.org/10.1038/s41588-024-01884-7

Genetic architecture of telomere length in 462,666 UK Biobank whole-genome sequences

In the format provided by the authors and unedited

Table of Contents

**SUPPLEMENTARY FIGURES**	**2**
**SUPPLEMENTARY TABLE LEGENDS**	**14**
**SUPPLEMENTARY METHODS**	**19**
**SUPPLEMENTARY NOTES**	**22**
**SUPPLEMENTARY REFERENCES**	**31**

Supplementary Figures

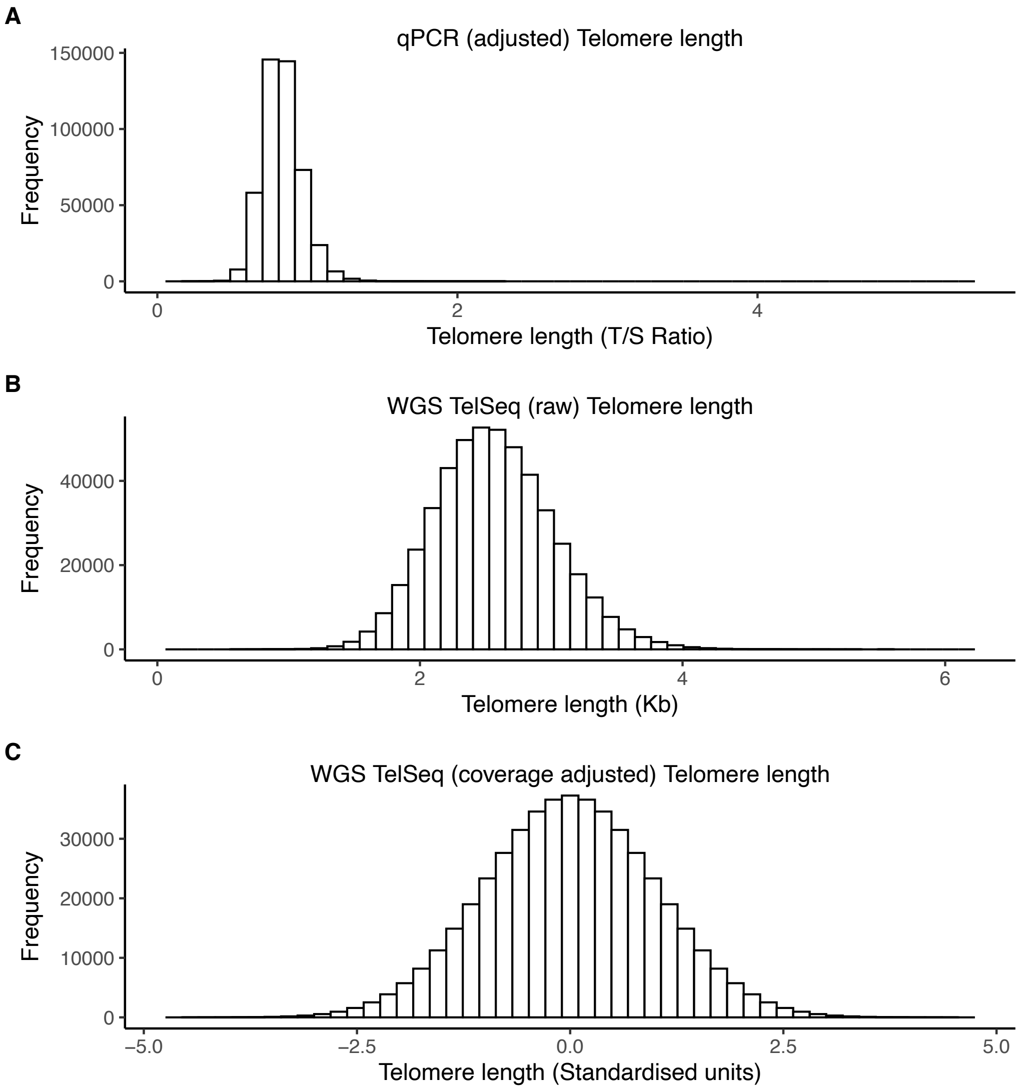

Supplementary Figure 1: Distribution of Telomere Length from different measurement platforms and adjustments. (A) qPCR adjusted T/S ratios (n= 462,666) released by *Codd et al.* (B) WGS TelSeq telomere length (n=482,839) across 482,839 participants. (C) coverage adjusted transformed WGS TelSeq telomere length estimates.

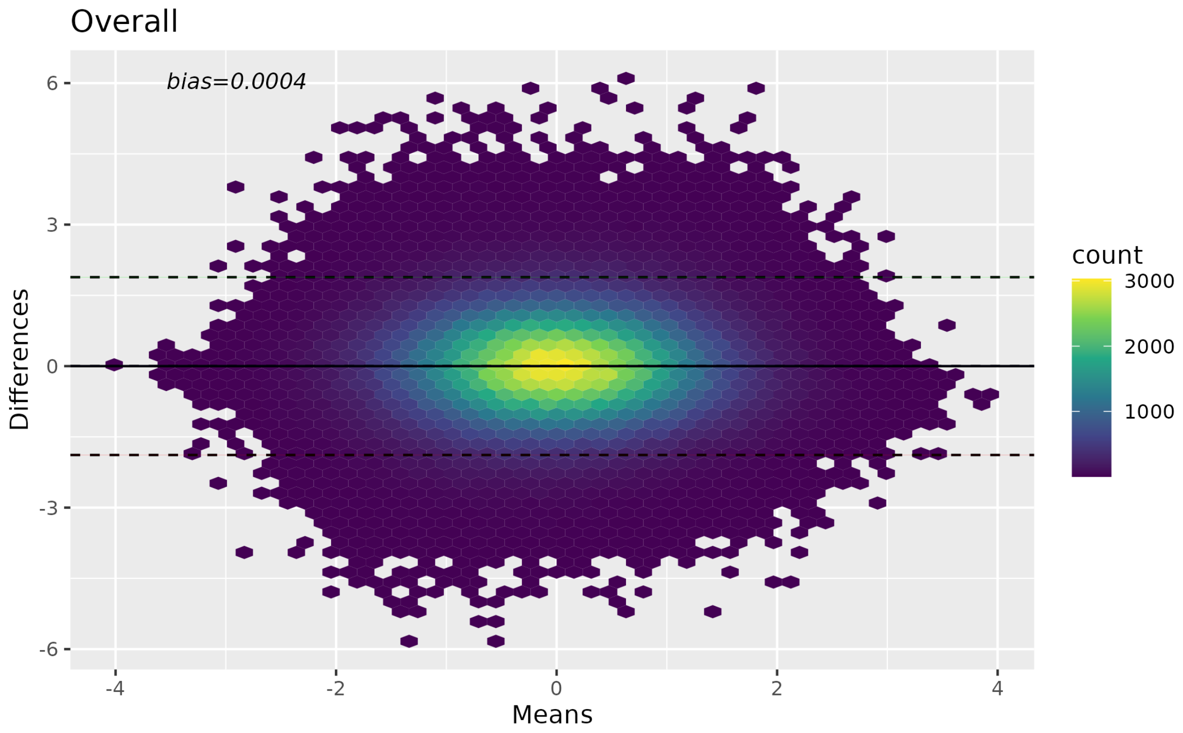

Supplementary Figure 2 : Bland-Altman plots comparing overall coverage adjusted TelSeq and qPCR telomere length estimates. To facilitate comparison at the same scale both metrics were inverse normal rank transformed before plotting. The black dashed lines from top to bottom represent 1.96 SD, mean and -1.96 SD respectively. Green, blue, and red shading indicates 95% confidence intervals associated with each of these. The bias (the difference between the expected difference i.e. 0) and the observed mean is shown in the top right corner. A positive value on the y-axis indicates a longer qPCR telomere length estimate for a participant than coverage corrected WGS TelSeq telomere length metric.

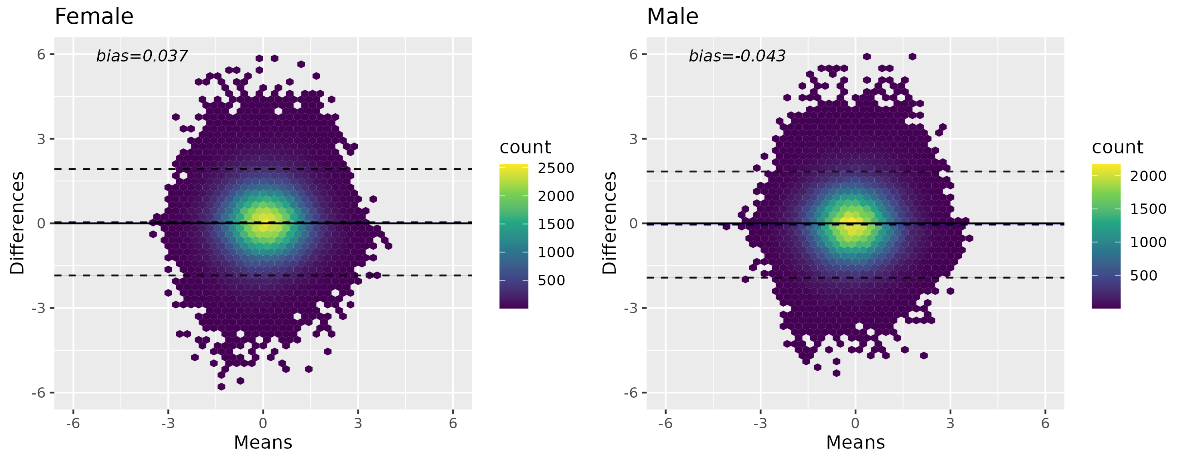

Supplementary Figure 3: Bland-Altman plots comparing coverage adjusted TelSeq and qPCR telomere length estimates by sex. The legends are the as for Supplementary figure 2.

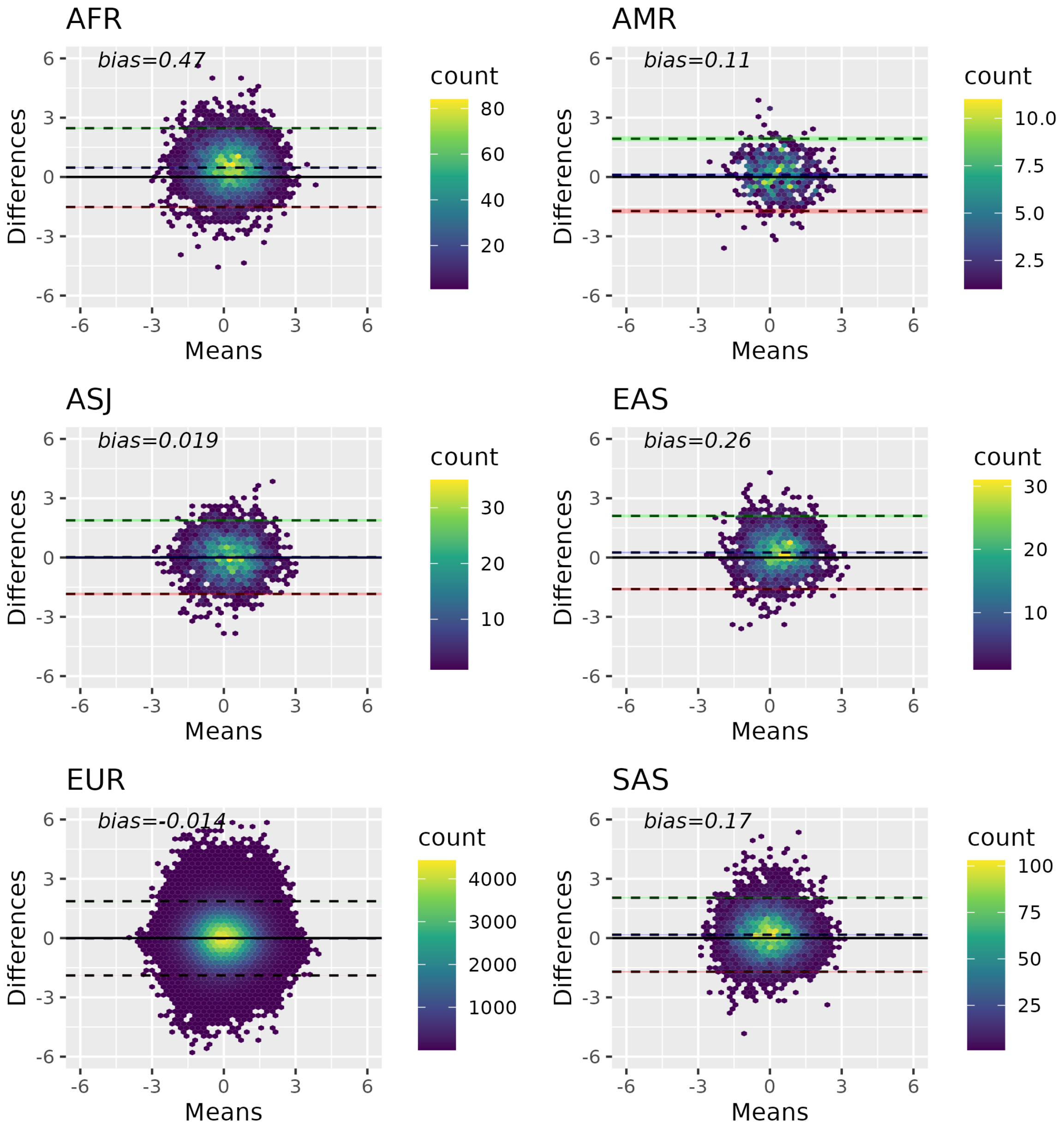

Supplementary Figure 4: Bland-Altman plots comparing coverage adjusted TelSeq and qPCR telomere length estimates by ancestry. The legends are the as for Supplementary figure 2.

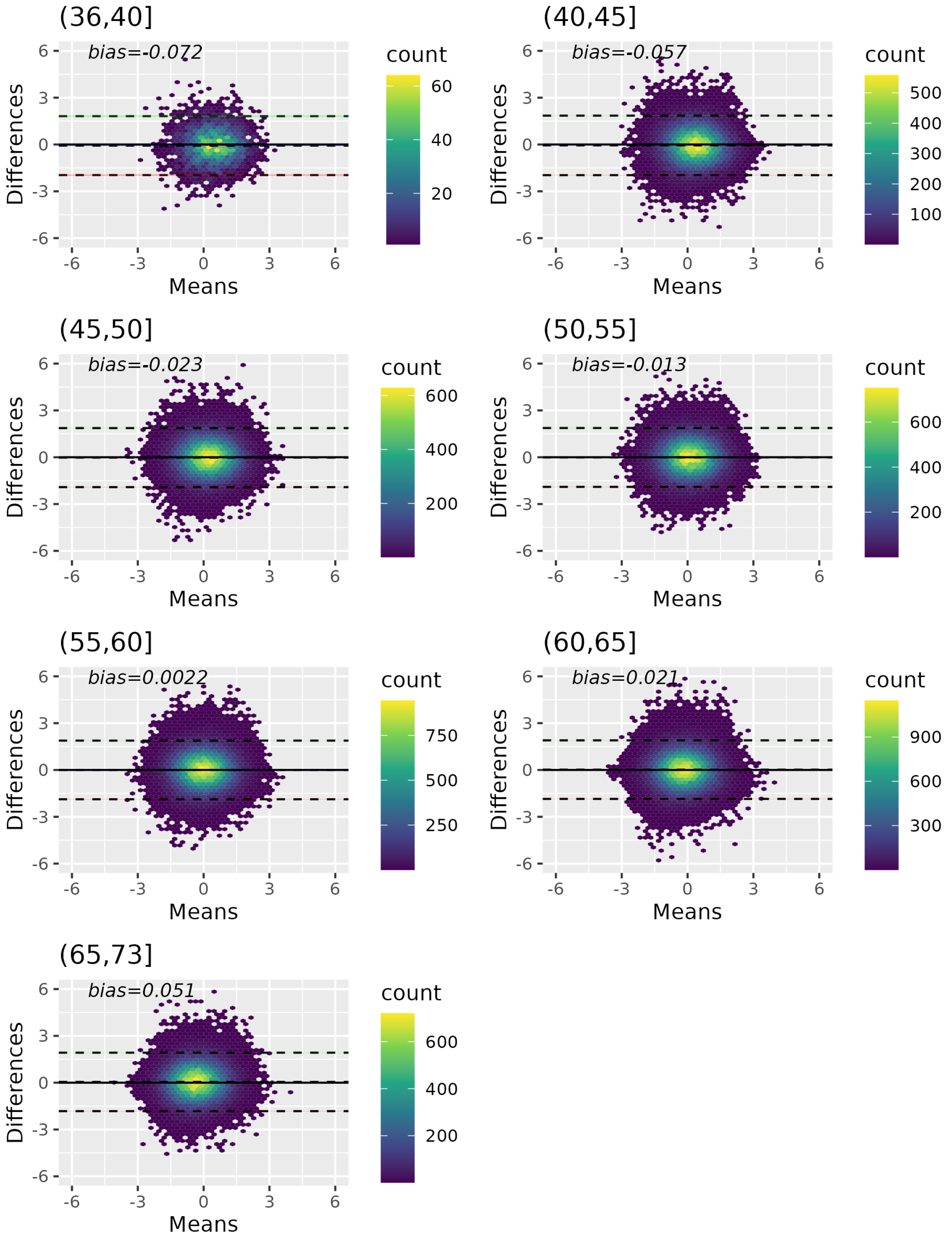

Supplementary Figure 5: Bland-Altman plots comparing coverage adjusted TelSeq and qPCR telomere length estimates by age strata. The legend is as for Supplementary figure 2.

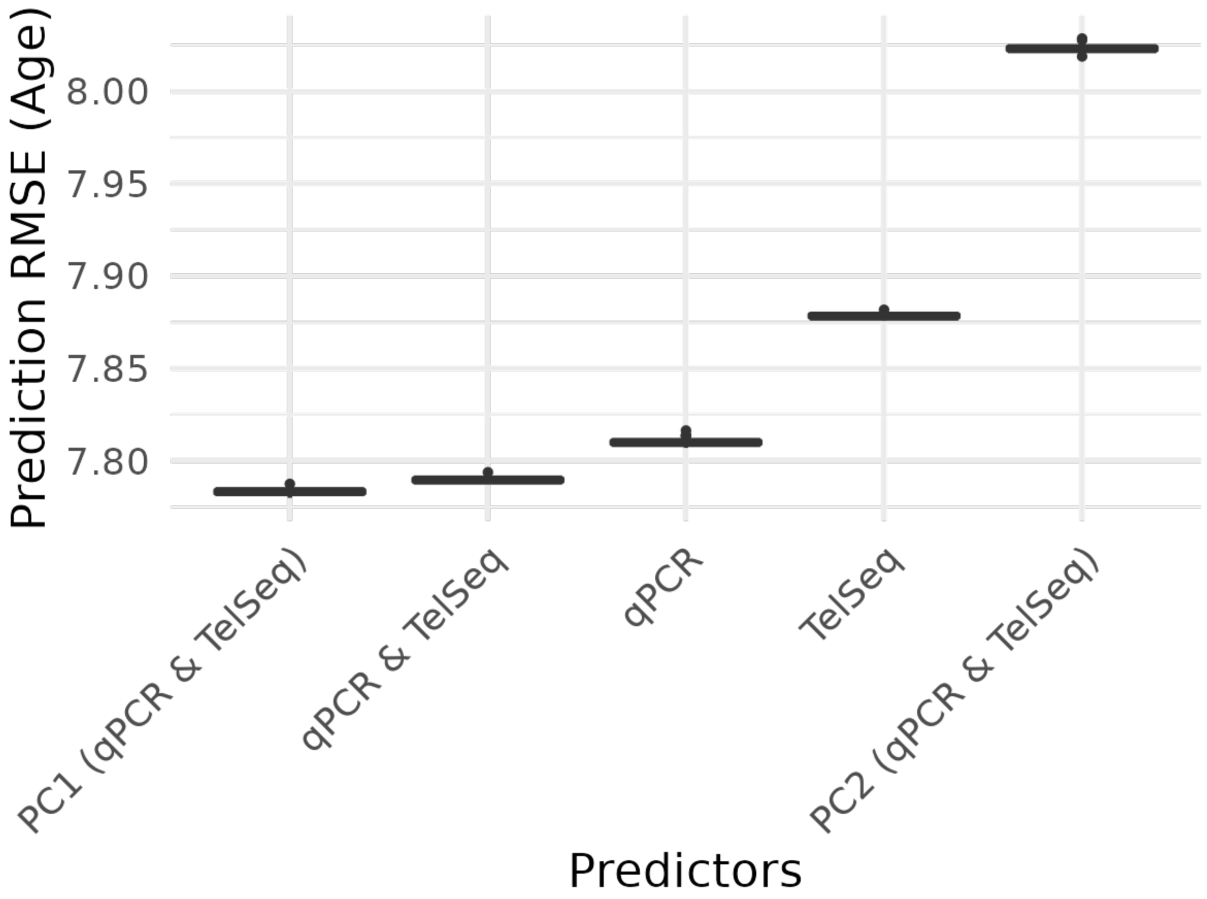

Supplementary Figure 6: Box plot of age prediction for different telomere length metrics. PC1/ PC2 (qPCR & TelSeq) indicates PCA derived composite score while qPCR & TelSeq indicates the joint model (i.e age ~ TLqPCR + TLTelSeq). Y-axis indicates root mean squared error (RMSE) of age prediction using a training set of 10,000 samples and applying the resultant model to the remaining held out samples, distributions were derived from carrying out this procedure 100 times (Methods). For each boxplot the centre is the median, the lower and upper hinges indicate the 25th and 75th percentile and outliers are represented as individual points.

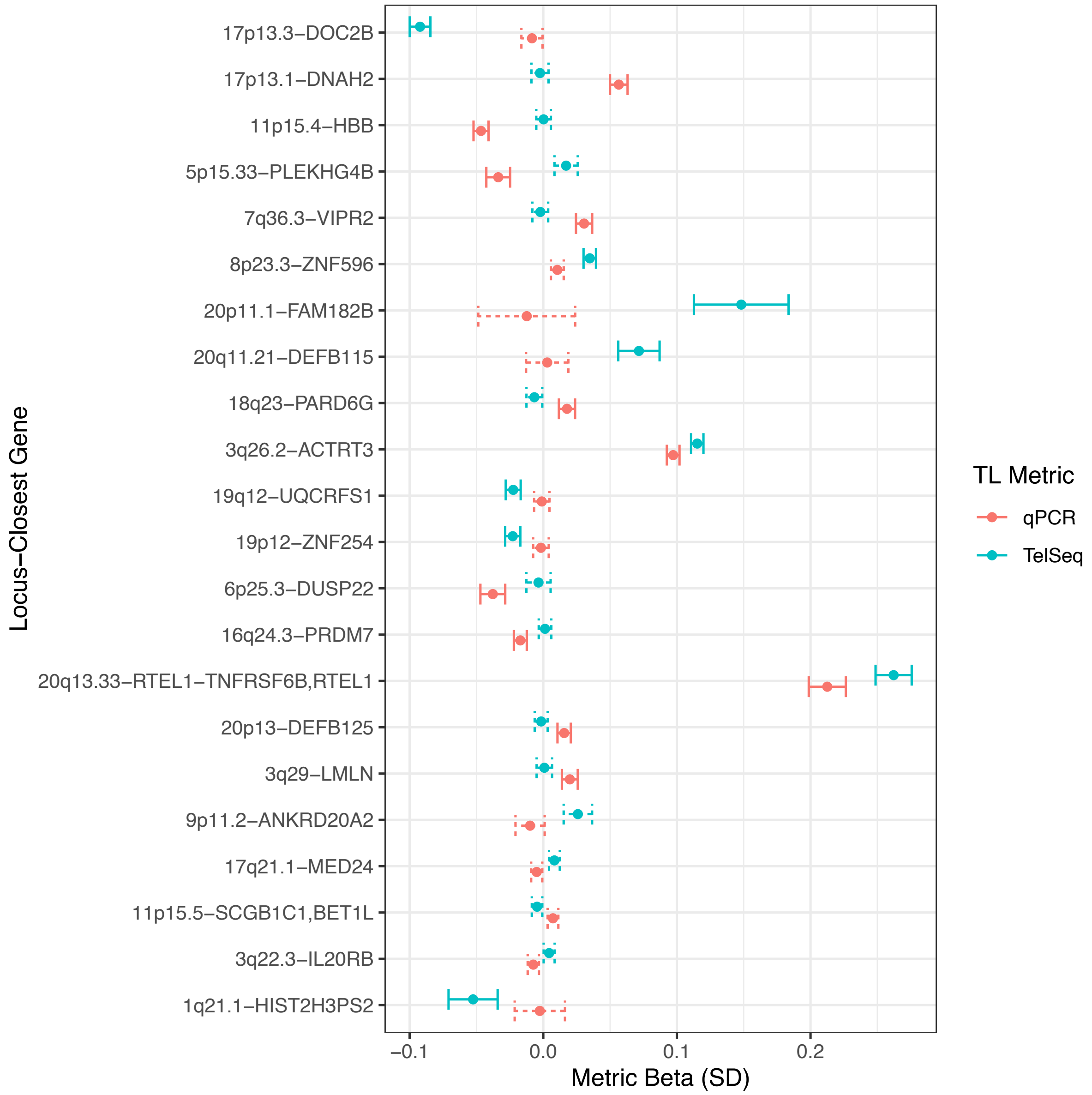

Supplementary Figure 7: Forest plot of qPCR and TelSeq association statistics for significant PC2 loci in NFE broad ancestries. Association statistics are derived using REGENIE for qPCR and WGS TelSeq (n=438,351 independent samples). Points indicate scaled telomere (TL) length metric effect size, with errpr bars representing 95% confidence intervals, dashed error bars indicate that the association was not genome-wide significant (p<5 x 10-8 two-sided unadjusted). Y axis is ordered by PC2 significance. PC2 is driven mainly by associations that differ between qPCR and TelSeq and may indicate spurious findings. We note that PC2 associations at 3q26.2 and 20q13.33 are associated in the same direction for both metrics and therefore are likely to constitute true associations albeit with non-overlapping effect sizes.

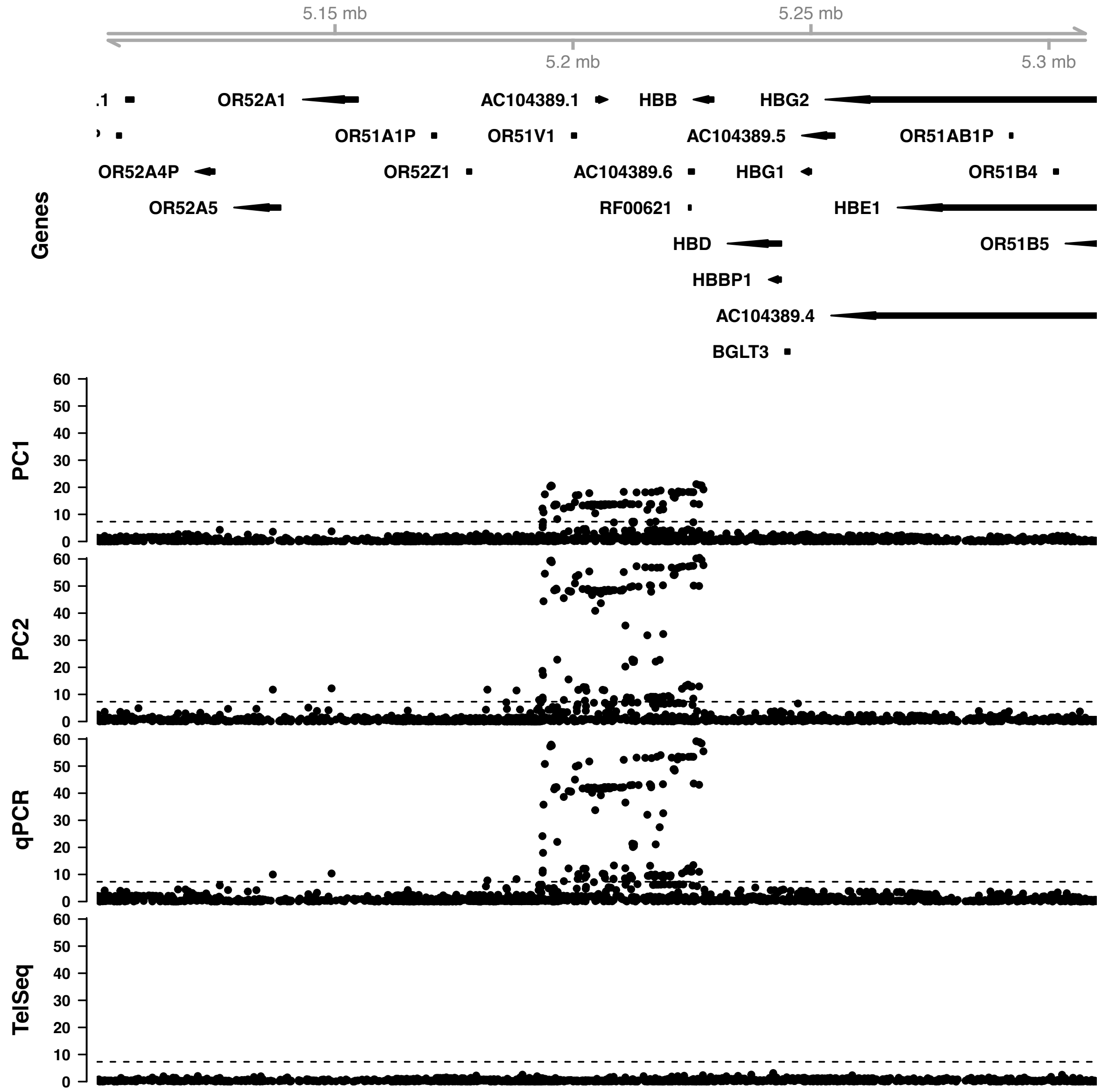

Supplementary Figure 8: Comparison of GWAS summary statistics for *HBB*/11p15.5 association. Coordinates are for GRCh38, stanza show -log10(p) derived from REGENIE regression (unadjusted two-sided p-values) for different telomere length metrics as labelled on the y-axis, dotted line indicates P=5 x 10^{-8}.

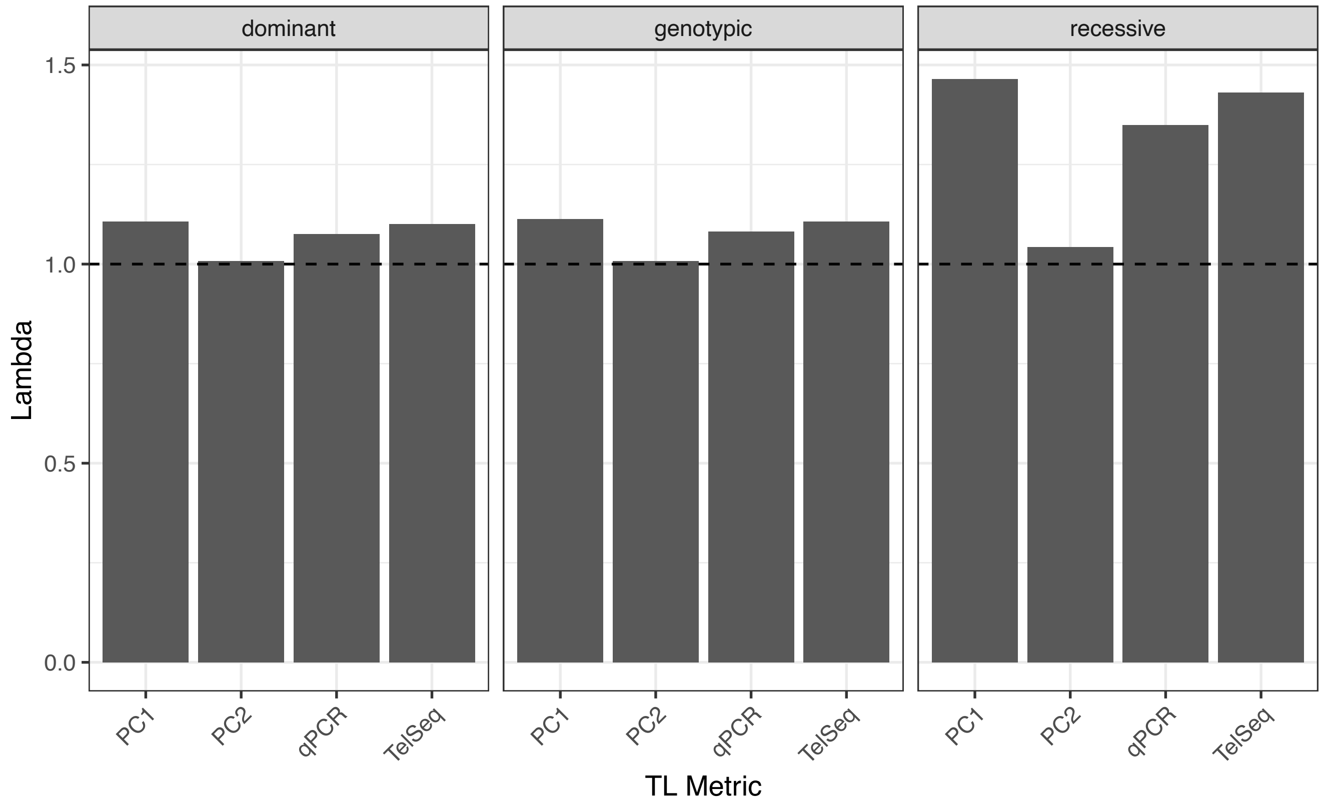

Supplementary Figure 9: Genomic control across ExWAS models. y-axis indicates lambda genomic control showing that generally inflation is well controlled for genotypic and dominant models. There is some evidence for inflation in the recessive model however no significant associations were reported for this model.

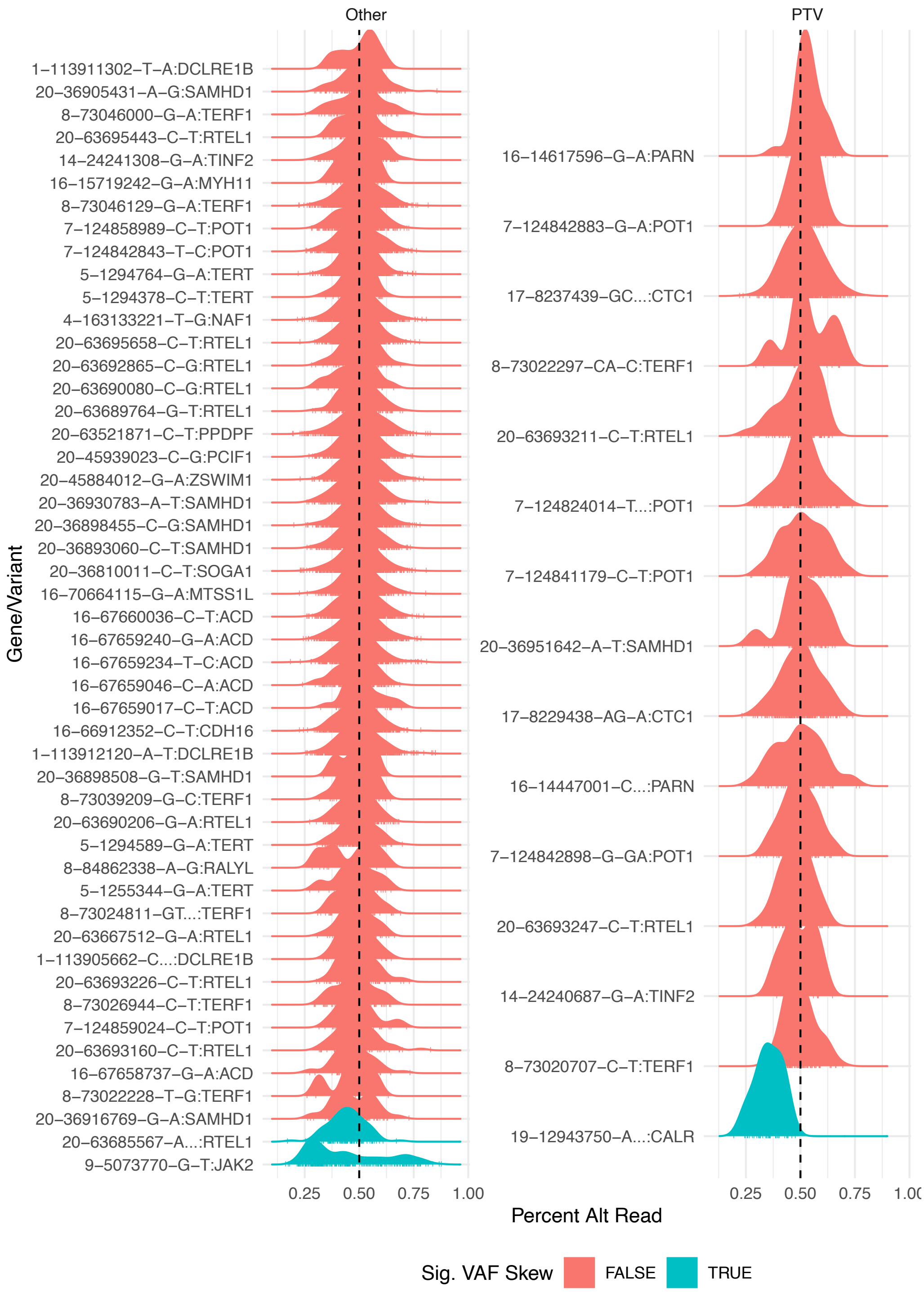

Supplementary Figure 10: Density rug ridge plot of percentage of alternative allele reads for heterozygous individuals for PC1 ExWAS associations that also don't overlap a PC2 ExWAS association. Sig variant allele fraction (VAF) skew indicates variants that when combined across individuals show significant departure from expected ratio of 1:1 (horizontal dotted line) for alt and ref reads assessed by a binomial test. The rug ends (lines) indicate the percentage of alternative allele reads for heterozygous of individuals.

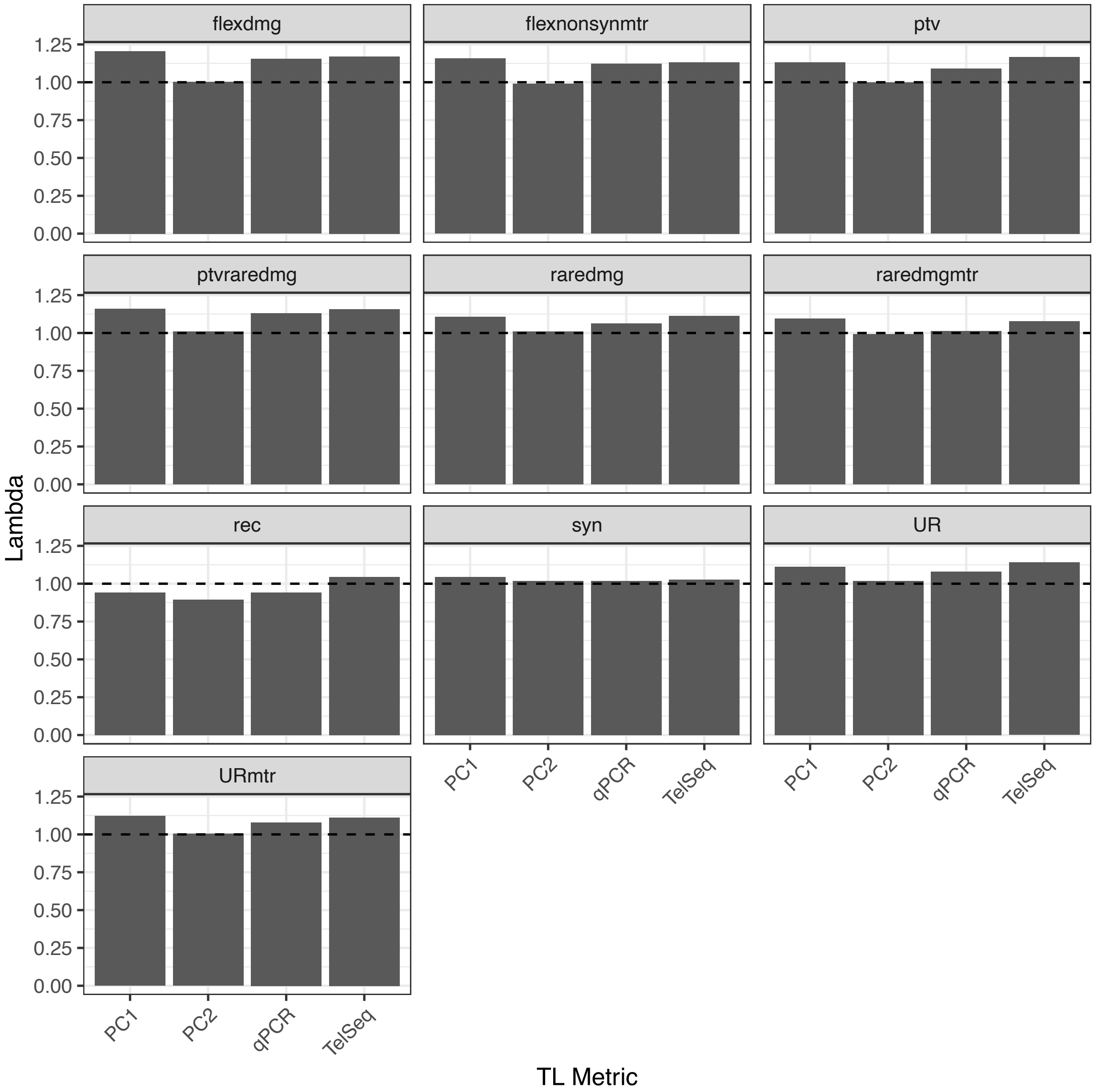

Supplementary Figure 11: Genomic control across 10 qualifying variant collapsing models. Y-axis indicates lambda genomic control showing that inflation is well controlled across all models.

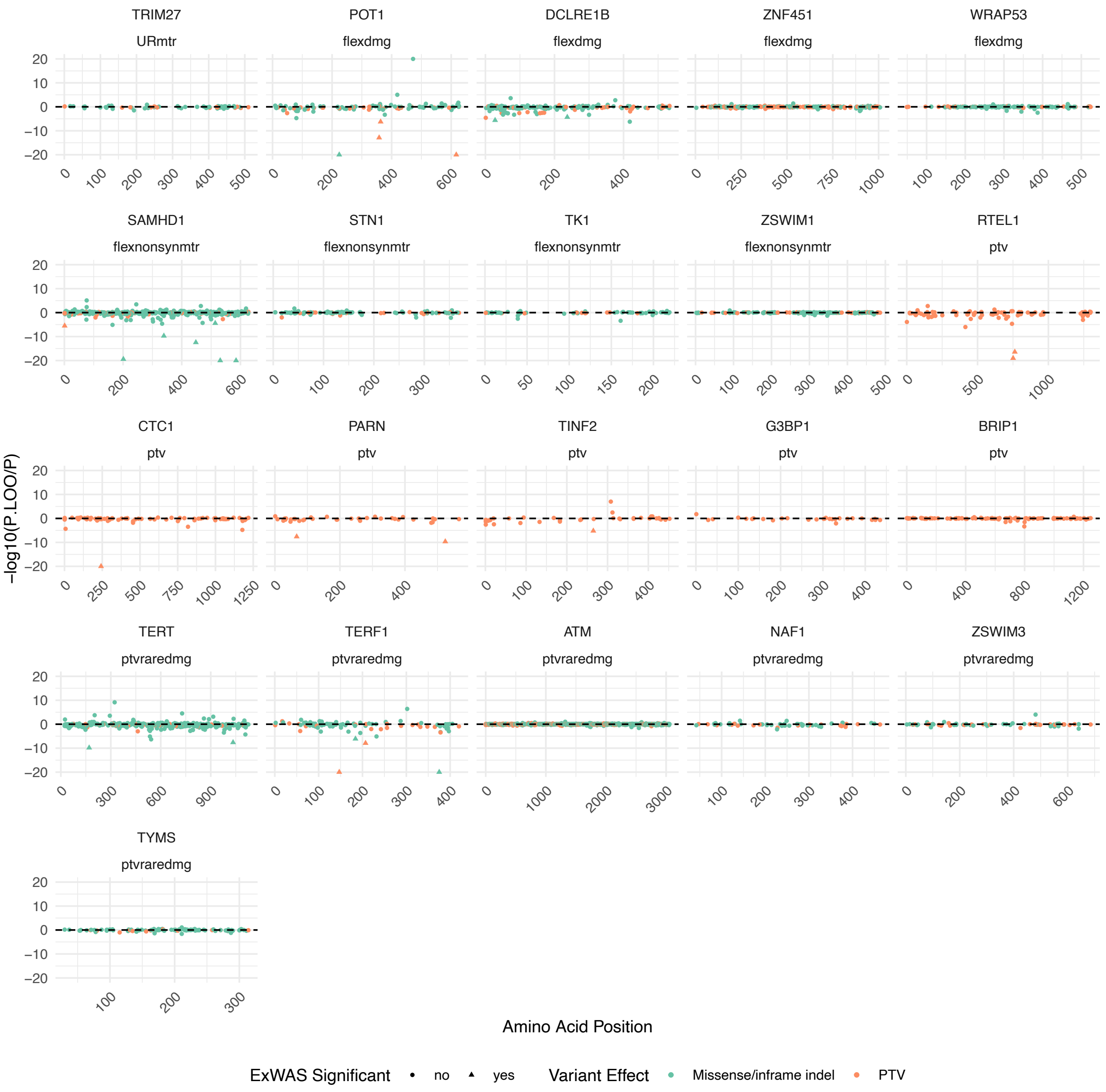

Supplementary Figure 12: Leave-one-out collapsing analysis of significant genes for most significant qualifying variant model. Points indicate variant left out and are coloured by function. Amino acid positions are based on canonical transcripts, triangular points indicate variants also found to be significant in ExWAS analyses. For clarity axes are truncated at -20 and 20.

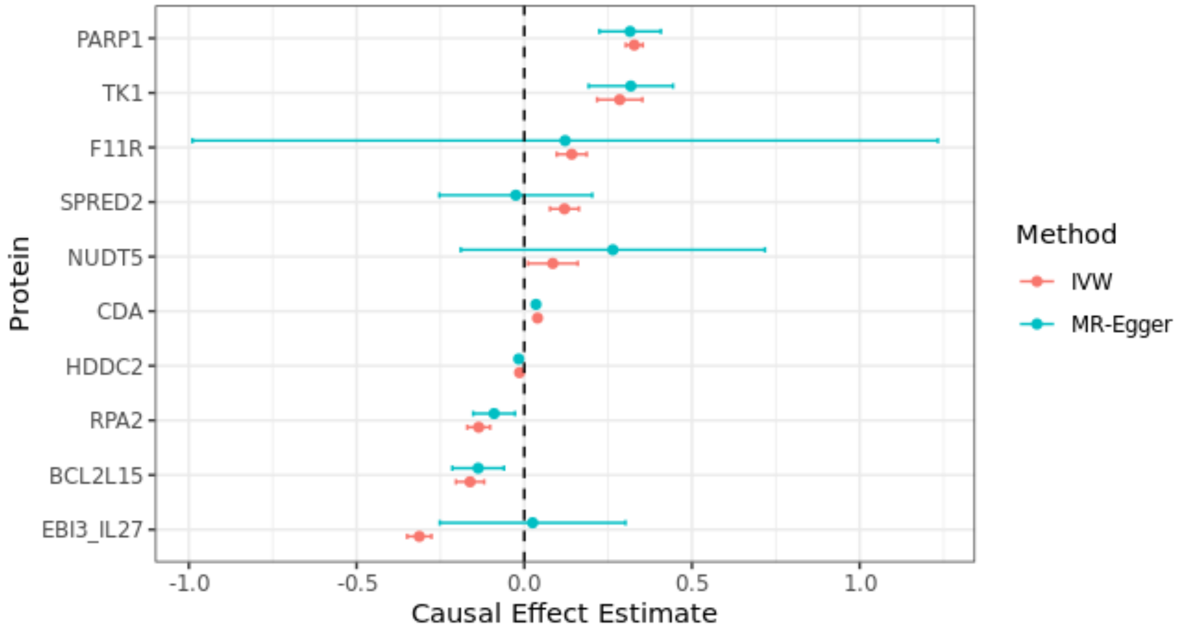

Supplementary Figure 13: Forest plot of causal effect estimates of cis colocalising pQTL proteins from UKB-PPP and Telomere length. Point estimates and error bars representing 95% confidence intervals are shown for inverse-weighted variance (coral) and MR-Egger approaches (azure). PPP GWAS summary statistics were obtained from Sun et al. discovery set (N=34,557 independent UKB samples), Telomere length GWAS summary statistics were taken from this study (N=462,666 independent UKB samples).

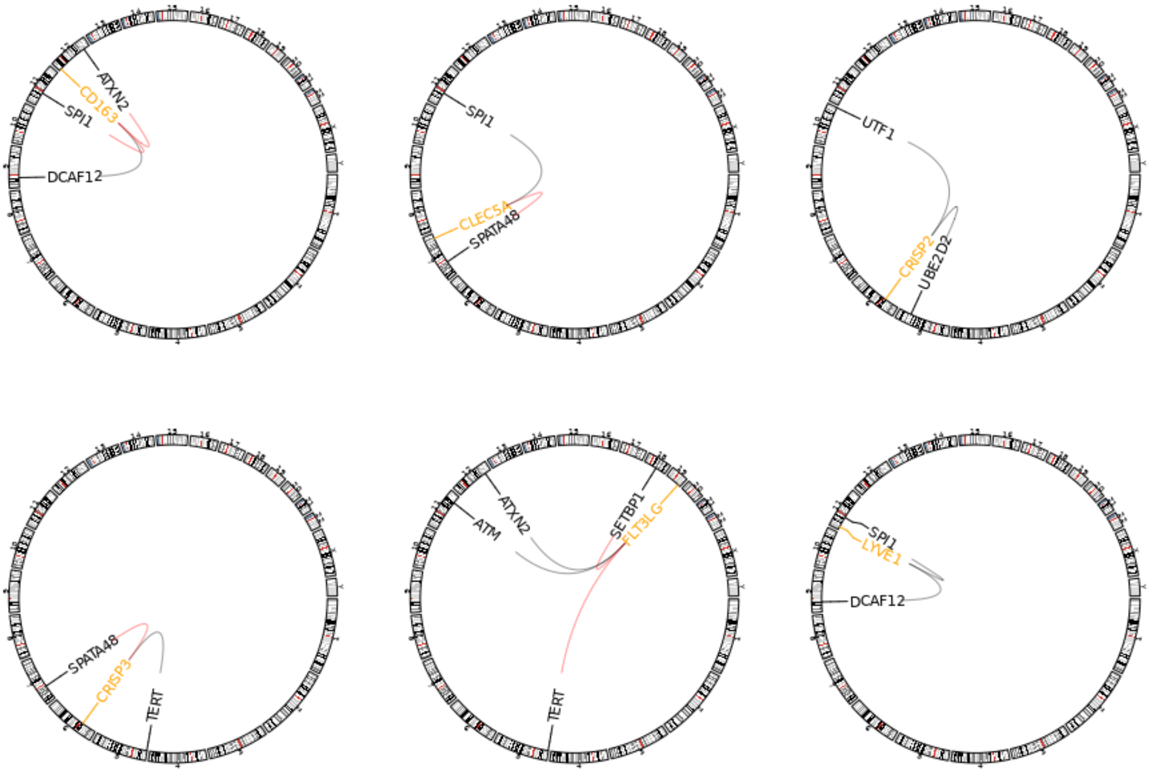

Supplementary Figure 14: Trans pQTLs for the same UKB PPP protein colocalising with multiple TL signals. Position of gene encoding the protein assayed is labelled in orange, whereas TL regions are labelled in black by putative causal prioritised or closest gene, a black connector indicates that effect sizes for protein and TL are the same direction (i.e. that increase in TL is associated with an increase in protein abundance), opposing effect directions are marked in with red connectors.

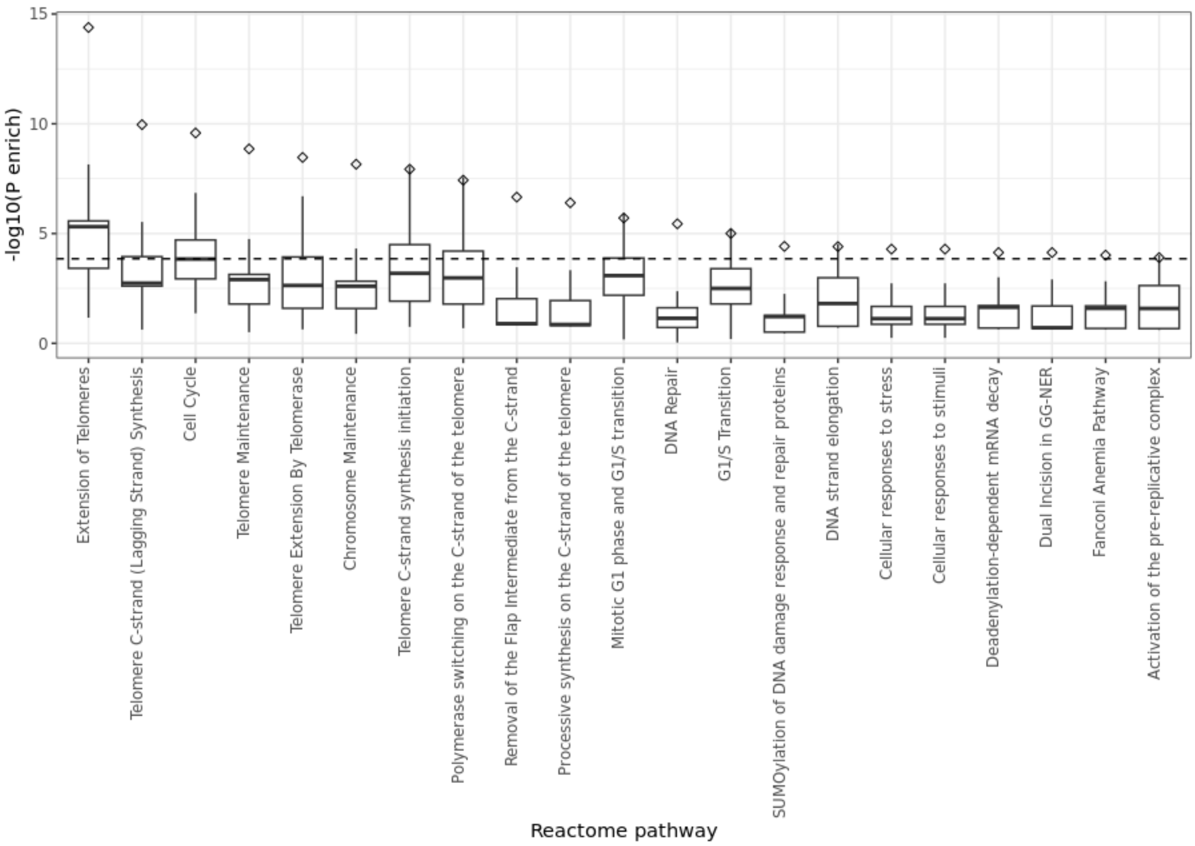

Supplementary Figure 15: Reactome pathway enrichment of prioritised genes for PC1 GWAS loci. Box plots (solid line median, hinges 25th and 75th percentile, whiskers indicate maximum and minimum values) representing -log10(p) enrichment were calculated using two-sided, unadjusted Fisher's exact tests across 50 sets of genes (N=94) randomly selected from those closest to the index variant (Methods). Diamonds show the actual -log10(p) enrichment for the prioritised gene set (N=94), using two-sided, unadjusted Fisher's exact test. The dashed line represents multiple testing p-value threshold.

Supplementary Table Legends

Supplementary Table 1: WGS Cohort Details: Ancestry denotes broad genetic ancestry classes as defined by 1000 genomes/gnomAD studies (AFR = African, AMR = Admixed American/Hispanic ASJ = Ashkenazi Jewish, EAS = East Asian, NFE = Non-Finnish European, SAS = South Asian). Telseq sample count indicates UKB participants with WGS data for which telomere length has been estimated using TelSeq. qPCR TL estimate indicates participants with telomere length estimates derived from qPCR (see Codd et al.).

Supplementary Table 2: Demographic Telomere length associations: Associations between age, sex and ancestry by telomere length metric (TelSeq and qPCR) for 462,666 independent participants. We fit the model TL ~ age + sex + ancestry where TL is the either the inverse normal rank transformed qPCR or TelSeq ancestry estimate, for each term in the joint model the p-value (two-sided, unadjusted), beta coefficient and 95% confidence intervals are shown. For sex and ancestry, coefficients are with respect to baseline Male or NFE participants respectively.

Supplementary Table 3: WGS Technical Associations: We fit a linear model to combined (PC1 and PC2) as well as individual telomere length metrics that included various WGS QC metrics as predictors as well as age and sex as positive controls to 462,666 independent samples. Non-categorical predictors metrics were inverse rank normal transformed before fitting to facilitate comparison so *_beta columns are approximately on the standard deviation scale. P-values are two sided, columns labelled *_p.adj are Bonferroni adjusted P-values.

Supplementary Table 4: Telomere length LD score regression. Results from LD score regression for 438,351 independent participants of broad NFE ancestry. Test statistics (two-sided, unadjusted) derived from *ldsc* software.

Supplementary Table 5: Telomere length GWAS Index variants. Index variants from GWAS of telomere length using imputed data for 438,351 independent participants of broad NFE ancestry. Summary statistics for these index variants for other ancestries are also shown (AFR = African (N=8,154), AMR = Admixed American/Hispanic (N=672), ASJ = Ashkenazi Jewish (N=2,629), EAS = East Asian (N=2,360), SAS = South Asian (N=9,286)). Effect sizes are with respect to each additional copy of A1 allele, Test statistics (two-sided, unadjusted) are derived from REGENIE. 'Distance to Telomere' indicates the number of base pairs from index variant to the nearest telomere (using GRCh38 coordinates from UCSC genome browser) and 'New Locus' whether a locus has been previously described (0=FALSE,1=TRUE). Finally we define a 'Locus ID' a unique numerical identifier to facilitate lookup/merging between other relevant Supplementary Tables.

Supplementary Table 6: COJO analysis of PC1 & PC2. Conditional analysis using GCTA COJO for PC1 and PC2 significant ($p<5 \times 10^{-8}$) loci. Effect size/beta is with respect to each additional copy of A1 allele. Test statistics (two-sided, unadjusted) are derived from COJO analysis of GWAS summary statistics on 438,351 independent participants of broad NFE ancestry.

Supplementary Table 7: PC1 & PC2 Telomere length rare variant (ExWAS) associations. Associations reaching P<=1e-8 for rare (MAF<1e-3) across ~436,410

independent participants of broad NFE are shown for PC1 and PC2 metrics. Test statistics (two-sided, unadjusted) are derived from fitting a linear model. Columns 'Gene Name', 'Most Damaging Effect' and Consequence are derived from snpEff. The most significant association across genotypic, dominant and recessive models is shown. Variants showing evidence for being somatic are marked in the 'somatic' column. 'GWAS Region' column indicates whether the rare variant overlaps a telomere length GWAS region. 'p_hwe' indicates deviation from Hardy-Weinberg equilibrium from a Chi-Squared test (two-sided, unadjusted). 'Has a minor homozygote carrier' indicates which variants have one or more homozygous carriers (0=FALSE,1=TRUE).

Supplementary Table 8: ExWAS conditional analysis. Results of pairwise conditioning of all ExWAS associated variants with each other across ~436,410 independent participants of broad NFE for PC1. Test statistics (two-sided, unadjusted) are derived from a linear model. 'Variant 1 P` is the P-value for Variant 1 association with telomere length. 'Variant 1 P conditional of Variant 2' is the P-value after conditioning on Variant 2. 'log(P) - log(conditional.P)' is the difference on the log scale between univariate and conditional P-values, and higher values indicate associations are not independent.

Supplementary Table 9: Gene collapsing qualifying variant models.

Supplementary Table 10: PC1 & PC2 Telomere length rare variant gene collapsing associations. Gene collapsing rare variant associations reaching P<=1e-8 across ~436,410 independent participants of broad NFE for PC1 and PC2 telomere length metrics. Test statistics (two-sided, unadjusted) are derived from fitting a linear model. Models are described in Supplementary Table 9. The 'Previous gene level association' column indicates whether a previous rare variant association between a gene and telomere length has been described. Associations showing evidence for being driven by somatic variants are marked in the 'Somatic' column.

Supplementary Table 11: Colocalisation analysis between UKB PPP pQTLs and PC1 Telomere length associations. Results of colocalization analysis between PC1 telomere length GWAS performed on 438,351 independent participants of broad NFE ancestry and UKB PPP pQTL summary statistics. Test statistics (two-sided, unadjusted) are derived from REGENIE, asymptotic Bayes Factors (*.abf) are derived from *coloc*. PPP.assay indicates the protein assay and 'index.variant.distance' is the distance in base pairs between the closest telomere length and pQTL index variant for that assay. 'coloc.nsnps' is the number of variants matching between PPP and telomere length summary GWAS stats that could be used by coloc. PPP.cis_trans indicates whether the PPP index variant is cis (within 1Mb of the gene encoding the protein being measured) or trans (further than 1Mb of the gene encoding the protein being measured). 'coloc.class' indicates evidence supporting colocalization between telomere length plasma protein level and is described fully in the methods. Note that the 'Locus ID' column refers to GWAS loci listed in Supplementary Table 5.

Supplementary Table 12: Plasma proteome Mendelian randomisation. Results from running Mendelian randomisation of plasma proteome instrumental variables on GWAS summary statistics for PC1 telomere length GWAS performed on 438,351

independent participants of broad NFE ancestry, for those gene-protein pairs exhibiting strong colocalization evidence for both a pQTL and telomere length associated variant. Test statistics (two-sided, unadjusted) are derived from MendelianRandomisation R package. Columns ‘Median P value overall and ‘Median adjusted P value overall’ denote the median P-value across all Mendelian randomisation methods and Bonferroni corrected median P-value respectively.

Supplementary Table 13: SuSIE Finemapping results. Results from running SuSIE fine-mapping framework on GWAS summary statistics for PC1 telomere length GWAS performed on 438,351 independent participants of broad NFE ancestry. Summary GWAS statistics (two-sided, unadjusted) are derived from REGENIE. `pip` and `cred.set` are the posterior inclusion probability for a variant to be causal and the causal set to which it belongs ascertained through SuSIE analysis. Note that the ‘Locus ID’ column refers to locus.id column in Supplementary Table 5.

Supplementary Table 14: GWAS Gene Prioritisation. Results from running gene prioritisation across all telomere length PC1 associated index variants from supplementary table 5 (438,351 independent participants of broad NFE ancestry). Gene Binary evidence columns are **Telomeropathy** - associated with a known telomeropathy from OMIM, ClinVar or HGMD, **Rare Variant**– associated with telomere length through NFE PC1 rare variant analyses (ExWAS and/or collapsing); **pQTL Colocalisation** – evidence for colocalisation between a UKB PPP pQTL and GWAS PC1 telomere length associations; **SuSiE Credset non-synonymous** – evidence from SuSIE analysis of GWAS PC1 telomere length associations of a credset containing a non-synonymous variant in the referenced gene; **Top PoPs score** – within the locus gene has top PoPs score; **Top ABC score** – within the locus gene has top Activity by contact (ABC) score and **Closest gene** – gene in locus closest to the index variant. **Prioritisation Score -** indicates the overall prioritisation score (the sum across all binary columns) for the referenced gene. **Prioritised in Locus** - indicates whether the gene within the locus that had the highest ‘Prioritisation score’ (note where multiple genes in the same locus all have the highest prioritisation score this column is set to ‘0’=FALSE). Finally, ‘abc.score’, ‘abc.tissue’ and ‘pops.score’ are the top Activity by contact (ABC) score, associated tissue and top PoPs scores respectively for the referenced gene. Note that the Locus ID column refers to Supplementary Table 5.

Supplementary Table 15: Prioritised genes Reactome enrichment. Results from Reactome enrichment analysis using prioritised GWAS genes (Supplementary Table 14). Test statistics are derived from ReactomePA (Fisher exact test - two-sided, undadjusted unless stated). **ID** - reactome pathway ID, **Prioritised Gene Ratio** – Total prioritised genes in pathway / Total prioritised genes. **Background Ratio –** Total genes in pathway not prioritised / Total ‘Universe of genes’ (i.e. all the protein-coding genes overlapping a locus). **Prioritised genes in pathway –** List of Gene Symbols for genes in tested Reactome pathway.

Supplementary Table 16: Cross ancestry Meta-analysis GWAS results for PC1 telomere length metrics. Additional index variants significantly associated with PC1 telomere length metric from a fixed effect inverse-variance weight meta-analysis

across all ancestries (AFR = African (N=8,154), AMR = Admixed American/Hispanic (N=672), ASJ = Ashkenazi Jewish (N=2,629), EAS = East Asian (N=2,360), NFE = Non-Finnish European (N=438,351), SAS = South Asian (N=9,286)). Test statistics are derived from METAL fixed effect IVW meta-analysis (two-sided, unadjusted). Closest gene and Distance to Telomere columns described in Supplementary Table 5.

Supplementary Table 17: Clonal Haematopoiesis Qualifying Variant Models.

Supplementary Table 18: PC1 Telomere Length associations with Clonal Haematopoiesis. Association of PC1 Telomere length with Clonal Haematopoiesis carrier status across 388,111 independent samples of broad NFE genetic ancestry. Test statistics (two-sided unadjusted) are derived from fitting a linear model. ‘Any’ indicates a model where an individual carries a CH QV any of the non VAF models described in Supplementary Table 17. N.CH.events indicates the total number of carriers for a given CH gene/QV model.

Supplementary Table 19: PC1 Telomere Length associations with Clonal Haematopoiesis stratified by Variant Allele Fraction (VAF). Association of PC1 Telomere length with Clonal Haematopoiesis carrier status stratified by VAF across 388,111 independent samples of broad NFE genetic ancestry. Test statistics (two-sided unadjusted) are derived from fitting a linear model. ‘Any’ indicates a model where an individual carries a CH QV in one or more of the VAF models described in Supplementary Table 17. N.CH.events indicates the total number of carriers for a given CH gene/QV model for a given VAF strata shown in the ‘Interval’ column.

Supplementary Methods

Causal gene prioritisation

To prioritise causal genes within NFE PC1 GWAS loci (**Supplementary Table 5**) we developed a prioritisation score based on seven equally weighted sources if information:

Genes implicated in a known telomeropathy.
We extracted genes with phenotypes containing ‘telomere’, ‘dyskeratosis congenita’, ‘hoyeraal-hreidarsson’, ‘revesz’ and ‘coats plus’ keywords across OMIM (2022-02-27) [https://omim.org/], HGMD[1] ‘DM’ (2023 Q2) and ClinVar[2] ‘Pathogenic’/’Likely Pathogenic’ (2023-12-03).

Rare variant association with telomere length
We extracted genes from our NFE rare variant analyses (**Supplementary Tables 7 and 10**) PC1 telomere length. From these we removed genes also associated with PC2 and shown to be driven by potential linkage disequilibrium with a neighbouring rare variant signal or driven by a somatic variant signal.

Telomere length signal colocalization with UKB PPP
As detailed in the main methods we took the list of genes with ‘strong’ evidence for colocalization (**Supplementary Table 11**) with one or more UKB PPP proteins in cis.

95% SusIE credible sets containing a missense or LoF variant: We annotated all variants falling within a SuSIE 95% credible set (**Supplementary Table 13**) using VEP[3] and then extracted all those genes overlapping a variant annotated as ‘HIGH’ or ‘MODERATE’ impact.

Activity by contact (ABC) score prioritisation: We used a similar approach as described in Nasser et al.[4] Briefly, we annotated all autosomal ABC regions excluding the MHC region as to whether they overlapped a variant falling within one or more 95% credible sets derived from SuSIE fine-mapping of PC1 telomere length GWAS (**Supplementary Table 13**) signals. We then performed Fisher’s exact test (two-sided) over each tissue type/context (n=131) to obtain a list of 21 tissue contexts that showed significant enrichment after Bonferroni multiple testing correction for putatively causal telomere length variants. From these we extracted ABC scores and linked target genes, taking forward the gene with the highest ABC score for a particular credible set.

PoPS score prioritisation: We followed the polygenic priority score (PoPS) approach described in Weeks et al.[5]. Briefly, we used MAGMA (v1.10)[6] to generate gene-level association statistics for our PC1 telomere length NFE GWAS statistics. We then applied PoPS, a method that assumes that causal genes share functional characteristics, that integrates data over 50,000 functional genomic features derived from gene expression, protein-protein interaction networks and pathway datasets. In total we generated PoPS scores for 18,383 protein coding genes. For each NFE GWAS locus we annotated the gene with the highest PoPS score in the locus.

Closest gene to index variant: We the Bioconductor library biomaRt to access Ensembl v92 gene annotations and selected the gene whose mid-point was closest to the index variant for NFE GWAS PC1.

In a similar manner to *Shrine et al.*[7] we created a list of 7,334 protein coding genes by intersecting telomere length PC1 NFE GWAS loci with gene locations from Ensembl (v92). For each of these genes we looked at overlap with each of seven feature sets described above; (1) implicated in a known telomeropathy, in HGMD, OMIM or Clinvar databases;(2) associated with telomere length in PC1, but not PC2 in exWAS or Collapsing rare variant analyses;(3) telomere length association signal colocalises in cis with a pQTL from UKB PPP [8];(4) SuSIE finemapping 95% credible set contained a missense/LoF variant (Supplementary Table 13); (5) Highest ABC target score across prioritised tissue contexts within an autosomal locus; (6) Highest PoPS score within an autosomal locus; (7) closest to index variant. We assumed equal weighting for each feature allowing us to derive an overall score as the sum of overlapping features for a given gene. For each locus we assigned a 'top prioritised gene', as the gene with the highest score.

To assess the relevance of the 'top prioritised gene' set (i.e. where for a given locus a gene is unambiguously prioritised), we performed gene set enrichment analysis of curated Reactome[9] pathway data using the 'ReactomePA' (v1.38.0) R package[10]. Briefly we remapped HGNC symbols to entrez_id's using the R Bioconductor package 'org.Hs.eg.db' v 3.14.0 for all 7,334 protein coding genes. We then used this 'universe' as the basis for enrichment analyses with 392 Reactome pathways where there were at least 10 'universe' genes in the pathway. We also performed a comparative analysis to assess the performance of using the closest gene. To do this we created 50 sets of genes of size 94 (matching the size of the 'top prioritised' gene set) by sampling with replacement from the set of genes closest to an index variant across PC1 GWAS loci. For each of these gene sets we performed gene set enrichment analysis using Reactome as described above to estimate the distribution of pathway enrichment statistics expected if a closest gene prioritisation strategy had been employed.

Supplementary Notes

Supplementary Note 1: on adjusting WGS TelSeq estimates for technical confounders.

Given the relatively low correlation between qPCR and WGS TelSeq TL estimates we sought to understand whether this could be due to various technical confounders. We captured 19 sequencing covariates for all 462,666 samples (**Supplementary Table 1**) where TL measurements were available for TelSeq and qPCR methods. Initially examined these variables in a univariate framework to understand which of these variables were significantly associated with either raw TelSeq, adjusted qPCR, or coverage adjusted TelSeq TL metrics. To do this we inverse normal transformed each TL outcome variables and then regressed each technical covariate in turn resulting 19 x 3 univariate associations (TelSeq, qPCR and PC1). From these we selected those technical variables that were Bonferroni significant (**Supplementary Table 3**). For comparison we also added age and sex as these have been previously associated in multiple published studies with TL. Overall (excluding age and sex) we detected 20 technical variables that on their own were significantly associated with at least one of the TL metrics (**Supplementary Fig. 8**). To understand the suitability of adjusting for these in our downstream analysis we performed three analyses. Firstly, we assessed the correlation between qPCR and coverage adjusted TelSeq TL without controlling for any of the technical variables identified above, using this as a baseline (r2=0.288). Using a linear model we next regressed out either all the significant technical variables or selected technical variables that were not themselves correlated with age or sex and repeated the correlation analysis. We found that adjusting for these technical confounders made little difference to qPCR correlation (all variables r2=0.273), (selected variables r2=0.288). For this reason, we decided to use just the baseline coverage TelSeq WGS in downstream analysis.

Supplementary Note 2: on optimal batch size to use when adjusting WGS TelSeq for coverage.

Due to computational constraints, it was necessary to perform coverage correction of WGS TelSeq TL estimates in batches. We performed analyses to ascertain the optimal size of these batches to minimise both batch effects and computational resource use. To do this we randomly selected 20,000 samples with both TelSeq and qPCR measurements. We then created 20 x 1K, 4 x 5K, 2 x 10K and 1 x 20K batches, which and then performed coverage correction as described in the main manuscript selecting 150 and 300 PCs. We also included an ‘adaptive’ benchmark where the number of PCs (to a max of 300) was selected to maximise the correlation. To benchmark correction success, we used the Pearson’s correlation coefficient between coverage adjusted TelSeq and qPCR TL measurements for each of the batches.

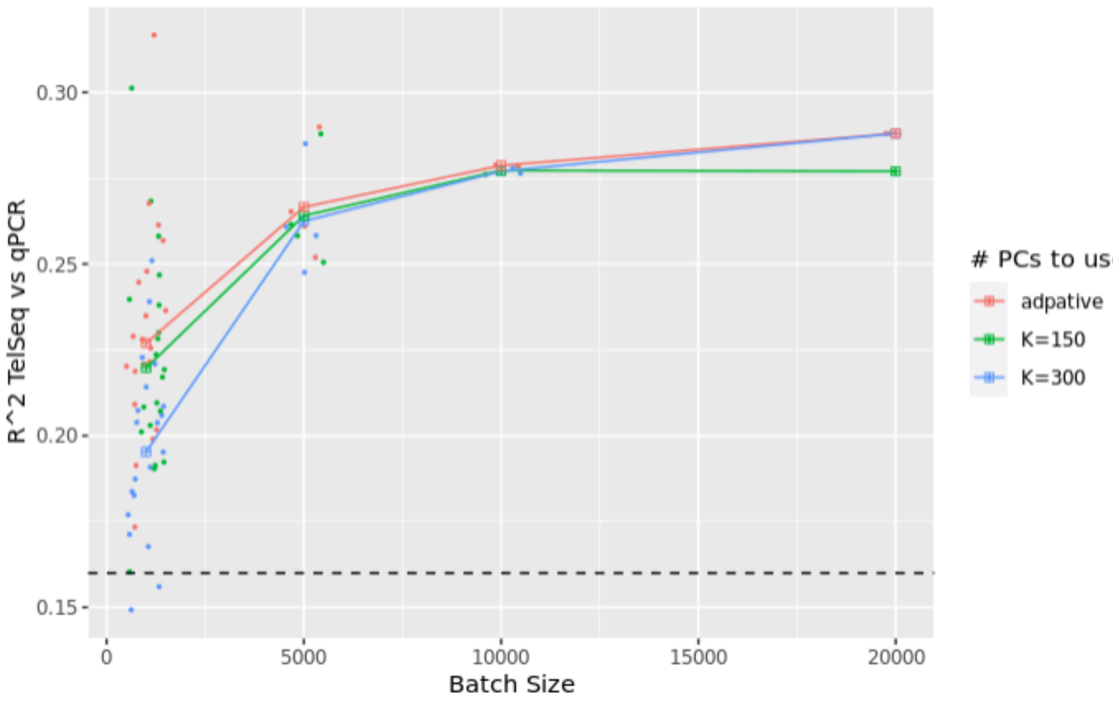

Supplementary Note 2, Figure 1: Effect of Batch and number of PCs on TelSeq TL finescale coverage correction performance. The x-axis shows batch sizes (20 x 1K, 4 x 5K, 2 x 10K and 1 x 20K) and the y the r^2 of coverage corrected TelSeq and qPCR TL estimates. Different colours indicate different number of PCs used to correct TelSeq TL, with adaptive (coral) indicating a non-fixed number of PCs as described above. The broken line indicates the correlation between qPCR and TelSeq TL with no coverage adjustment. The curves/points indicate the median correlation achieved for each condition.

As expected at small batch sizes there was significant variability in the correlation with qPCR after TelSeq correction, with smaller numbers of PCs giving better performance. As batch size increased there was a decrease in the number of batches although this was accompanied by a noticeable decrease in variability and an increase in correlation. As can be seen above though at larger batch sizes with more PCs the correlation with qPCR begins to plateau and concomitantly the memory footprint required to approximate the PCs increases exponentially. Due to resource constraints, we had access to a maximum of 0.2Tb of RAM and given the observed diminishing returns with larger batch sizes we took forward 24 (23 x 20K and one of 22,839) randomly sampled batches for coverage adjustment so as to balance computational resources with best performance. After coverage adjustment we did not find evidence of a measurable batch effect.

Supplementary Note 3: on GWAS comparison with *Codd et al.*

We compared our GWAS results with a previously published GWAS that used qPCR TL measurements on the same set of participants. As expected, effect sizes from our GWAS on qPCR, WGS, PC1 and PC2 TL were all highly correlated with their qPCR-based effect sizes (**Supplementary Figure 11**) and we were able to replicate associations reported as significant by *Codd et al.* ($p<8.3x10^{-9}$, N Loci=131) at 131, 81, 124 and 7 loci, across the respective TL measures (**Supplementary Table 5**).

Supplementary Note 4: on the effect of mosaic loss of chromosome X and Y on qPCR and WGS TelSeq TL estimates.

Mosaic loss of X (mLoX) or Y (mLoY) chromosomes could theoretically lead to differences in TL estimates between qPCR and TelSeq approaches, which could underlie some of the differences we observed between these metrics. In both metrics, the numerators are a measure of telomere content. However, for qPCR-derived estimates, the denominator is a measure of the abundance of the single copy gene (S) *HBB*, which is expected to be unaffected by mLoX/Y. For TelSeq, the denominator reflects the total GC adjusted WGS read count and is expected be attenuated in the event of significant mLoX/Y. Under these assumptions we might therefore expect shorter TL estimates from qPCR than TelSeq in the presence of mLoX/Y.

To test this, we considered 19 sentinal germline variants that were associated with LoY in a prior GWAS [11]. Of these 19, 11 were also significantly associated ($P_{Bonferroni} < 0.003$) with TL. Under the hypothesis that mLoY would bias estimates, we would expect to identify significantly different effect size estimates for qPCR versus TelSeq. However, there was no overall evidence of effect size heterogeneity between metrics (Table 1), suggesting that mLoY is not systematically biasing TL estimates.

Supplementary Note 4: Table 1 Results of Heterogeneity test (1 degree freedom two-sided χ^2 test), of effect sizes between TelSeq and qPCR TL effect estimates for variants also associated with mLoY, effect allele is a1. P heterogeneity shows raw significance whilst adjusted incorporates Bonferroni correction).

rs#	Variant (chr-pos-a0-a1)	Cochrane's Q	P heterogeneity	Adjusted P heterogeneity
rs13191948	6-109313396-T-C	4.56	0.02	0.18
rs4754301	11-108177814-A-G	3.17	0.04	0.41
rs13088318	3-101523907-G-A	2.89	0.04	0.49
rs381500	6-164057356-A-C	2.35	0.06	0.69
rs11082396	18-44500755-C-T	2.07	0.08	0.83
rs1122138	14-95713905-A-C	0.32	0.28	1.00
rs17758695	18-63253621-T-C	0.82	0.18	1.00
rs2736609	1-156232849-T-C	0.29	0.30	1.00
rs77522818	17-49740011-T-A	1.62	0.10	1.00
rs10687116	13-41103945-AGATG-A	0.50	0.24	1.00
rs115854006	3-48346680-T-C	0.08	0.39	1.00

At the individual variant level, we observed differing effect (Figure 1). For example, for rs11082396[18-44500755-C-T] the major allele (T) was associated with reduced mosaic Y loss ($beta_{LoY}$= 0.003) in Wright et al. but is associated with shorter TL for both qPCR and TelSeq. Altogether, these results demonstrate that mLoY is not a major source of bias in either approach to estimating TL and that mLoY-associated variants can have different effects on TL.

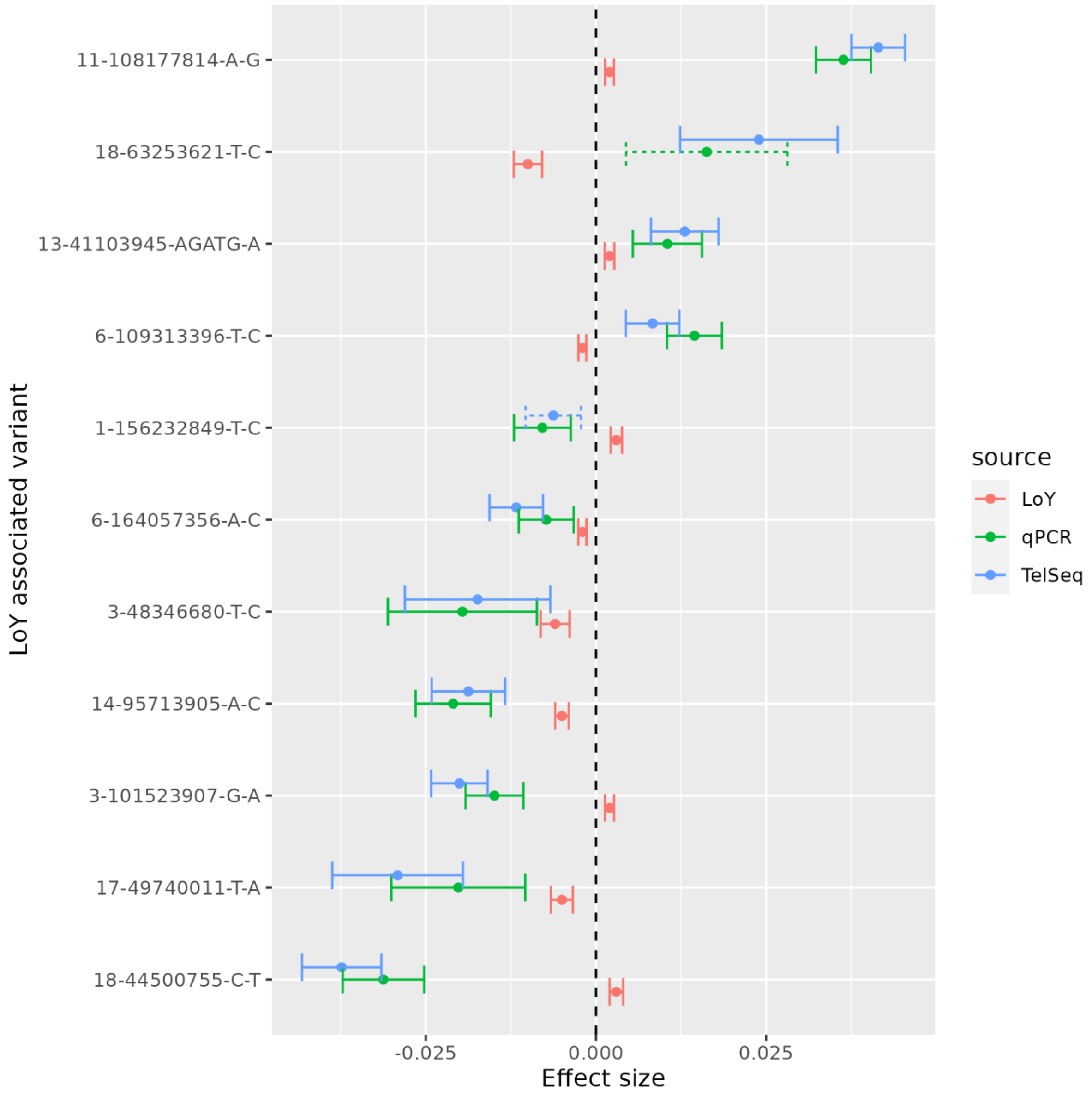

Supplementary Note 4, Figure 1 Forest plot of significant variants associated with mLoY (N = 67,034 independent male samples - coral) on a log(OR) scale from *Wright et al.* with matching effect sizes from qPCR (N = 462,666 independent NFE genetic ancestry samples - green) and TelSeq (N = 462,666 independent NFE genetic ancestry samples blue) SD scale. Error bars represent 95% confidence intervals. Variants are labelled chr-position-a0-a1 where a1 is the effect allele. p-values are two-sided and unadjusted.

In a second analysis, we used the mosaic chromosomal alteration (mCA) data returned to UK Biobank (Field ID 3094) from Loh et al. (Loh, Genovese, and McCarroll 2020) and followed the method described in Kessler et al. (Kessler et al. 2022) to create a binary indicator as to whether an UKB participant showed evidence of mosaic loss of X or Y. We used this to investigate whether there was an association between presence or absence of mLoX or mLoY and telomere length using a similar regression framework as described in the main manuscript to investigate clonal haematopoiesis (CH). Briefly,

we removed individuals with known haem malignancies or high lymphocyte counts (>5) as well as those individuals with a somatic CH driver variant. We then used a linear model to assess whether there was an association between either mLoY or mLoX in males and females, respectively, and either qPCR or TelSeq TL metrics adjusting for age, smoking status, and ancestry PCs. We did not include sex, or related covariates given the sex specific nature of the phenotypes.

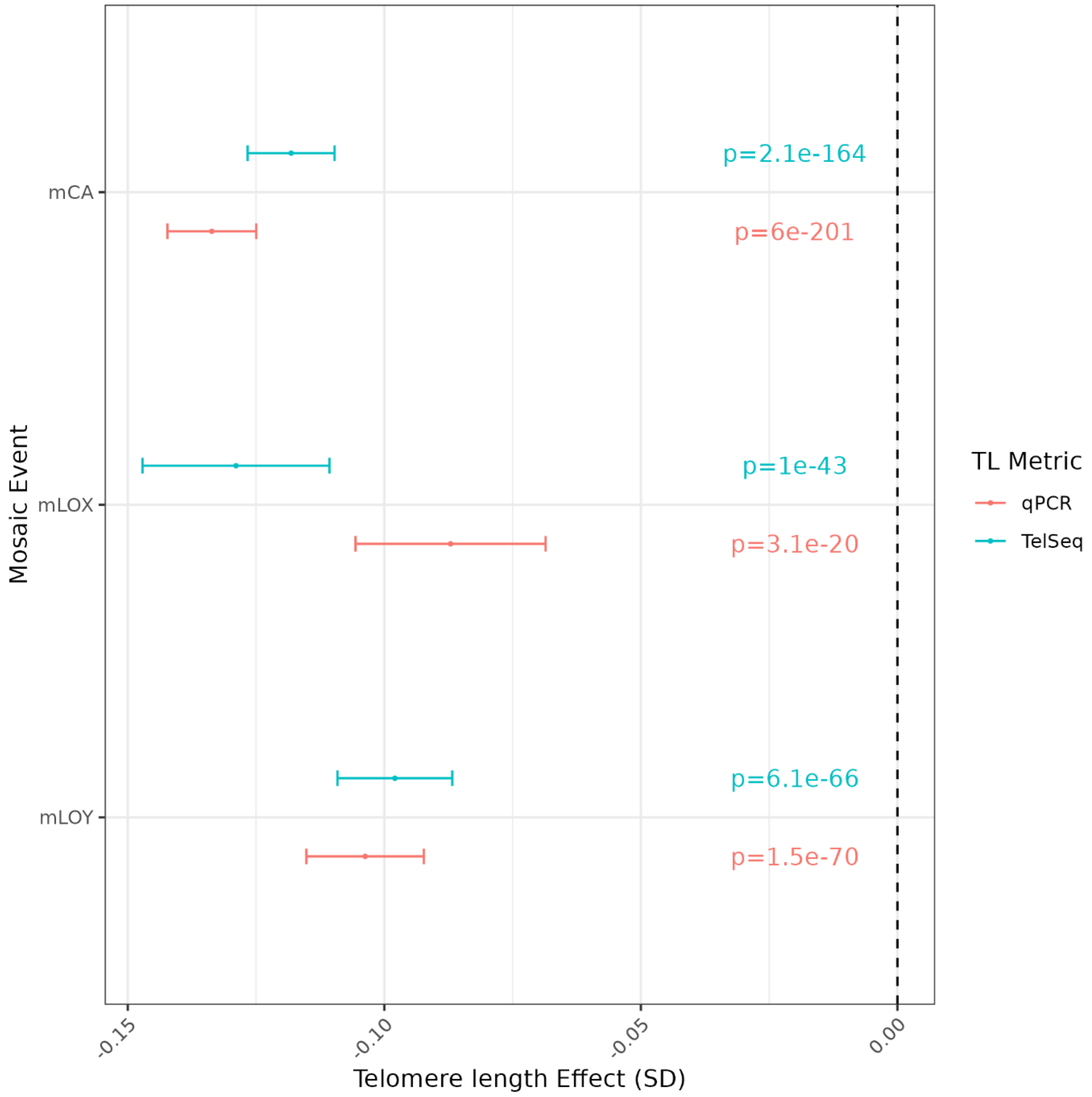

Supplementary Note 4, Figure 2 Association between mosaic chromosomal alterations and telomere length measurements. Telomere length measurements from qPCR and WGS TelSeq are shown in different colours. For mCA – mosaic chromosomal alterations (N = 418,865 independent samples) mLoX – mosaic loss of X (N= 227,520 independent female samples) and mLoY – mosaic loss of Y (N= 191,345 independent male samples). Telomere length effect estimates from fitting a linear model along with 95% confidence intervals are shown, p-values are two-sided and unadjusted.

Overall, we observed that all classes of mosaic chromosomal alteration (mCA), were associated with shorter TL. The effect sizes between metrics exhibited heterogeneity. For example, for overall mCA, the qPCR TL point effect size estimate was less/shorter than TelSeq, concordant with expectations of systematic differences between the metrics. However, for mLoX this was inverted, and TelSeq effect size point estimates were less/shorter.

Of relevance we note that [12] found no evidence for significant genetic correlation between qPCR LTL and mLOX/Y, which could be due to discordant effect sizes resulting in no net correlation. These results indicate a complex relationship between mLoX/mLoY and TL. Further studies will be required to fully understand the technical and biological mechanisms that underpin these observations.

Supplementary References

1. Stenson, P. D. *et al.* The Human Gene Mutation Database: building a comprehensive mutation repository for clinical and molecular genetics, diagnostic testing and personalized genomic medicine. *Hum. Genet.* **133**, 1–9 (2014).
2. Landrum, M. J. *et al.* ClinVar: public archive of relationships among sequence variation and human phenotype. *Nucleic Acids Res.* **42**, D980-5 (2014).
3. Hunt, S. E. *et al.* Annotating and prioritizing genomic variants using the Ensembl Variant Effect Predictor-A tutorial. *Hum. Mutat.* **43**, 986–997 (2022).
4. Nasser, J. *et al.* Genome-wide enhancer maps link risk variants to disease genes. *Nature* **593**, 238–243 (2021).
5. Weeks, E. M. *et al.* Leveraging polygenic enrichments of gene features to predict genes underlying complex traits and diseases. *Nat. Genet.* **55**, 1267–1276 (2023).
6. de Leeuw, C. A., Mooij, J. M., Heskes, T. & Posthuma, D. MAGMA: Generalized Gene-Set Analysis of GWAS Data. *PLoS Comput. Biol.* **11**, e1004219 (2015).
7. Shrine, N. *et al.* Multi-ancestry genome-wide association analyses improve resolution of genes and pathways influencing lung function and chronic obstructive pulmonary disease risk. *Nat. Genet.* **55**, 410–422 (2023).
8. Sun, B. B. *et al.* Plasma proteomic associations with genetics and health in the UK Biobank. *Nature* 1–10 (2023).
9. Milacic, M. *et al.* The reactome pathway knowledgebase 2024. *Nucleic Acids Res.* (2023) doi:10.1093/nar/gkad1025.
10. Yu, G. & He, Q.-Y. ReactomePA: an R/Bioconductor package for reactome pathway analysis and visualization. *Mol. Biosyst.* **12**, 477–479 (2016).

11. Wright, D. J. *et al.* Genetic variants associated with mosaic Y chromosome loss highlight cell cycle genes and overlap with cancer susceptibility. *Nat. Genet.* **49**, 674–679 (2017).

12. Kessler, M. D. *et al.* Common and rare variant associations with clonal haematopoiesis phenotypes. *Nature* **612**, 301–309 (2022).
