# Supplementary material for: Genetic architecture of telomere length in 462,666 UK Biobank whole-genome sequences
Source: Nat Genet. 2024 Aug 27;56(9):1832–40. doi: 10.1038/s41588-024-01884-7 (PMC11387196; doi:10.1038/s41588-024-01884-7)
Supplement: Supplementary file 2 — Reporting Summary [file 41588_2024_1884_MOESM2_ESM.pdf]

nature portfolio

Corresponding author(s): Slave Petrovski

Last updated by author(s): Jul 8, 2024

Reporting Summary

Nature Portfolio wishes to improve the reproducibility of the work that we publish. This form provides structure for consistency and transparency in reporting. For further information on Nature Portfolio policies, see our Editorial Policies and the Editorial Policy Checklist.

Statistics

For all statistical analyses, confirm that the following items are present in the figure legend, table legend, main text, or Methods section.

n/a	Confirmed	
☐	☒	The exact sample size (*n*) for each experimental group/condition, given as a discrete number and unit of measurement
☐	☒	A statement on whether measurements were taken from distinct samples or whether the same sample was measured repeatedly
☐	☒	The statistical test(s) used AND whether they are one- or two-sided<br>*Only common tests should be described solely by name; describe more complex techniques in the Methods section.*
☐	☒	A description of all covariates tested
☐	☒	A description of any assumptions or corrections, such as tests of normality and adjustment for multiple comparisons
☐	☒	A full description of the statistical parameters including central tendency (e.g. means) or other basic estimates (e.g. regression coefficient) AND variation (e.g. standard deviation) or associated estimates of uncertainty (e.g. confidence intervals)
☐	☒	For null hypothesis testing, the test statistic (e.g. *F*, *t*, *r*) with confidence intervals, effect sizes, degrees of freedom and *P* value noted<br>*Give P values as exact values whenever suitable.*
☒	☐	For Bayesian analysis, information on the choice of priors and Markov chain Monte Carlo settings
☒	☐	For hierarchical and complex designs, identification of the appropriate level for tests and full reporting of outcomes
☐	☒	Estimates of effect sizes (e.g. Cohen's *d*, Pearson's *r*), indicating how they were calculated

Our web collection on statistics for biologists contains articles on many of the points above.

Software and code

Policy information about availability of computer code

Data collection	Single-sample processing, on Amazon Web Services (AWS) cloud compute platform.<br>* Conversion of sequencing data in BCL format to FASTQ format and the assignments of paired-end sequence reads to samples based on 10-base barcodes; bcl2fastq v2.19.0 https:/ /support.illumina.com/sequencing/sequencing_software/bcl2fastq-conversion-software.html<br>* read alignment and variant calling performed on Illumina DRAG EN Bio-IT Platform Germline Pipeline v3.0.7 to align the reads to the GRCh38 genome reference [http://ftp.1000genomes.ebi.ac.uk/vol1/ftp/technical/reference/GRCh38_reference_genome/.] and perform small variant SNV and indel calling. SNVs and indels were annotated using SnpEFF v4.3 against Ensembl Build 38.92. We further annotated all variants with their gnomAD minor allele frequencies (gnomAD v2.l.1 mapped to GRCh38).<br>* For ancestry, we used PEDDY v0.4.2 with the ancestry labeled 1K Genomes Project reference sequence data for genetic ancestry predictions.<br>* For relatedness, we used ukb_gen_samples_to_remove() function from the R package ukbtools v0.11.3.
Data analysis	* WGS Telomere length estimation was performed using TelSeq v0.0.2 (https://github.com/zd1/telseq)<br>* PheWAS and exWAS association tests were performed using a custom built frame PEACOK (PEACOK 1.0.7), which is an extension and enhancement of PHESANT. PEACOK 1.0.7 can be found: https://github.com/astrazeneca-cgr-publications/PEACOK/versions/1.0.7<br>* GWAS was performed using REGENIE v3.1 (https://rgcgithub.github.io/regenie/)<br>* LD score regression was performed using LDSC v1.01 (https://github.com/bulik/ldsc)<br>* Approximate conditional association was performed using GCTA/COJO v1.94.1 (https://yanglab.westlake.edu.cn/software/gcta/#Download)<br>* Genotype data management and LD pruning was performed using PLINK v1.9 (https://www.cog-genomics.org/plink/) and PLINK v2.0 (https://www.cog-genomics.org/plink/2.0/)<br>* To call somatic CH variants we used Mutect2 v.4.2.2 (https://gatk.broadinstitute.org/hc/en-us/articles/4405443657499-Mutect2)<br>* Large-scale compute was done using AWS Batch computing environment.

* We used genome sequence-derived genotypes for biallelic autosomal SNVs located in coding regions as input to the kinship algorithm included in KING v2.2.3.
* We use PLINK1 (v1.90b6.21) and PLINK2 (v2.00) for genotype processing and LD pruning.
* MAGMA v1.08 to integrate functional data to prioritise putative causal genes.
* PoPS v 0.2 to integrate functional data to prioritise putative causal genes.
* susieR v 0.12.35 library to perform finemapping
* QCTOOLS v2.0.6 and BCTOOLS v1.11 to manage genotype data
* Various downstream analysis and summarization were performed using R v4.1.0 https://cran.r-project.org. R library MASS (7.3-51.6), pacman (0.5.1), data.table (v 1.14.0) tidyverse (2.0.0) ggplot2 (v3.4.4) rtracklayer (1.54.0), GenomicRanges (1.46.1), cowplot (1.1.3), patchwork (1.2.0), biomaRt (2.5.3), ggrepel (0.9.5) and ukbtools (v0.11.3)

For manuscripts utilizing custom algorithms or software that are central to the research but not yet described in published literature, software must be made available to editors and reviewers. We strongly encourage code deposition in a community repository (e.g. GitHub). See the Nature Portfolio guidelines for submitting code & software for further information.

Data

Policy information about availability of data

All manuscripts must include a data availability statement. This statement should provide the following information, where applicable:

- Accession codes, unique identifiers, or web links for publicly available datasets
- A description of any restrictions on data availability
- For clinical datasets or third party data, please ensure that the statement adheres to our policy

Full summary association statistics generated in this study will be publicly available through our AstraZeneca Centre for Genomics Research (CGR) PheWAS Portal (http://azphewas.com/) or GWAS catalog (https://www.ebi.ac.uk/gwas/) [GCST90435144 & GCST90435145]. All whole-genome sequencing data and qPCR data described in this paper are publicly available to registered researchers through the UKB data access protocol. Genomes can be found in the UKB showcase portal: https://biobank.ndph.ox.ac.uk/showcase/label.cgi?id=100314. qPCR-derived TL estimates are available at https://biobank.ndph.ox.ac.uk/ukb/label.cgi?id=265, and WGS TelSeq estimates will be made available as a 'Returned Dataset' . Additional information about registration for access to the data is available at http://www.ukbiobank.ac.uk/register-apply/. Data for this study were obtained under Resource Application Numbers 26041 and 68601.

Research involving human participants, their data, or biological material

Policy information about studies with human participants or human data. See also policy information about sex, gender (identity/presentation), and sexual orientation and race, ethnicity and racism.

Reporting on sex and gender	All analyses included males and females. We report that sex was used as a covariate in the association analyses.
Reporting on race, ethnicity, or other socially relevant groupings	94% of the cohort is of European ancestry.
Population characteristics	The average age was 57, and 54% of the cohort was female
Recruitment	Participants were recruited to the UK Biobank on a voluntary basis. Approx 500K individuals 40-69 years of age in 2006-2010 volunteered. Informed consent was obtained for all participants. It has previously been observed that participants are less likely to live in socioeconomically deprived areas than non-participants, and they tend to be healthier than non-participants, which may impact some of the reporting rates in comparison to what could be observed through random sampling from the UK population.<br>Fry et al (10.1093/aje/kwx246).
Ethics oversight	The protocols for UK Biobank are overseen by The UK Biobank Ethics Advisory Committee (EAC), for more information see https://www.ukbiobank.ac.uk/ethics/ and https://www.ukbiobank.ac.uk/wp-content/up1oads/2011/05/EGF20082.pdf

Note that full information on the approval of the study protocol must also be provided in the manuscript.

Field-specific reporting

Please select the one below that is the best fit for your research. If you are not sure, read the appropriate sections before making your selection.

☒ Life sciences ☐ Behavioural & social sciences ☐ Ecological, evolutionary & environmental sciences

For a reference copy of the document with all sections, see nature.com/documents/nr-reporting-summary-flat.pdf

Life sciences study design

All studies must disclose on these points even when the disclosure is negative.

Sample size	There were 490,560 UKB participants with WGS data. We further subset the cohort based on QC metrics as described in the manuscript. No sample size calculations for power were performed.

Data exclusions	At the sample level, we excluded samples based on predefined exclusion criteria as detailed in the manuscript. Briefly, we excluded those that did not pass sequencing quality control thresholds.
Replication	We replicated the signals from our GWAS with signals from a prior GWAS performed on the same cohort (see Supplementary Note) and was not independent.
Randomization	This study is observational. Randomization was not applicable to this study.
Blinding	This study is observational, using coded de-identified data. Blinding was not applicable to this study.

Reporting for specific materials, systems and methods

We require information from authors about some types of materials, experimental systems and methods used in many studies. Here, indicate whether each material, system or method listed is relevant to your study. If you are not sure if a list item applies to your research, read the appropriate section before selecting a response.

Materials & experimental systems

n/a	Involved in the study	
☒	☐	Antibodies
☒	☐	Eukaryotic cell lines
☒	☐	Palaeontology and archaeology
☒	☐	Animals and other organisms
☒	☐	Clinical data
☒	☐	Dual use research of concern
☒	☐	Plants

Methods

n/a	Involved in the study	
☒	☐	ChIP-seq
☒	☐	Flow cytometry
☒	☐	MRI-based neuroimaging
